# Combination analysis of genome-wide association and transcriptome sequencing of residual feed intake in quality chickens

**DOI:** 10.1186/s12864-016-2861-5

**Published:** 2016-08-09

**Authors:** Zhenqiang Xu, Congliang Ji, Yan Zhang, Zhe Zhang, Qinghua Nie, Jiguo Xu, Dexiang Zhang, Xiquan Zhang

**Affiliations:** 1 Guangdong Provincial Key Lab of Agro-Animal Genomics and Molecular Breeding and Key Lab of Chicken Genetics, Breeding and Reproduction, Ministry of Agriculture, Guangzhou, 510642 Guangdong Province China; 2 Wen’s Nanfang Poultry Breeding Co. Ltd, Guangdong Province Yunfu, 527400 China

**Keywords:** Residual feed intake, Genome-wide association study, RNA-sequencing, Quality chickens

## Abstract

**Background:**

Residual feed intake (RFI) is a powerful indicator for energy utilization efficiency and responds to selection. Low RFI selection enables a reduction in feed intake without affecting growth performance. However, the effective variants or major genes dedicated to phenotypic differences in RFI in quality chickens are unclear. Therefore, a genome-wide association study (GWAS) and RNA sequencing were performed on RFI to identify genetic variants and potential candidate genes associated with energy improvement.

**Results:**

A lower average daily feed intake was found in low-RFI birds compared to high-RFI birds. The heritability of RFI measured from 44 to 83 d of age was 0.35. GWAS showed that 32 of the significant single nucleotide polymorphisms (SNPs) associated with the RFI (*P* < 10^−4^) accounted for 53.01 % of the additive genetic variance. More than half of the effective SNPs were located in a 1 Mb region (16.3–17.3 Mb) of chicken (*Gallus gallus*) chromosome (GGA) 12. Thus, focusing on this region should enable a deeper understanding of energy utilization. RNA sequencing was performed to profile the liver transcriptomes of four male chickens selected from the high and low tails of the RFI. One hundred and sixteen unique genes were identified as differentially expressed genes (DEGs). Some of these genes were relevant to appetite, cell activities, and fat metabolism, such as *CCKAR*, *HSP90B1,* and *PCK1*. Some potential genes within the 500 Kb flanking region of the significant RFI-related SNPs detected in GWAS (i.e., *MGP*, *HIST1H110*, *HIST1H2A4L3*, *OC3*, *NR0B2*, *PER2*, *ST6GALNAC2*, and *G0S2*) were also identified as DEGs in chickens with divergent RFIs.

**Conclusions:**

The GWAS findings showed that the 1 Mb narrow region of GGA12 should be important because it contained genes involved in energy-consuming processes, such as lipogenesis, social behavior, and immunity. Similar results were obtained in the transcriptome sequencing experiments. In general, low-RFI birds seemed to optimize energy employment by reducing energy expenditure in cell activities, immune responses, and physical activity compared to eating.

**Electronic supplementary material:**

The online version of this article (doi:10.1186/s12864-016-2861-5) contains supplementary material, which is available to authorized users.

## Background

Residual feed intake (RFI) is an effective indicator for the feed utilization efficiency of animals. Low RFI selection enables a lower energy intake without sacrificing growth performance because it is independent of the metabolic body weight and daily gain regardless of the phenotype or genetic level [1, 2]. The feed conversion rate (FCR) is another synthetic trait that can optimize energy usage and is negatively correlated with growth traits [3, 4]. Chinese consumers prefer quality chickens (also called yellow plumage chickens) with a long feeding time and excellent meat quality but focus less on body weight at the market. Therefore, RFI selection is more applicable to non-fast growing chickens for the genetic improvement of energy metabolism.

To date, up to 60 quantitative trait loci (QTLs) have been reported to have significant associations with chicken feeding traits including dry matter digestibility, dry matter intake, feeding efficiency, feed intake, RFI and FCR, of which 20 were associated with RFI (http://www.animalgenome.org/QTLdb/). However, the above RFI-related QTLs are difficult to be applied to quality chickens due to the differences on breeds and trait measurement time.

Genome-wide association studies (GWAS), at first, are used to screen candidate markers associated with human diseases [5, 6]. With the continuous improvement and reduced cost of genotyping technology, this technique is being more widely applied to animal breeding and genetics. GWAS-related applications in RFI are primarily concentrated on beef cattle [7–9] and pigs [10–12] and have been applied to laying hens [13] and broilers [14]. The development of the high density 600 K genotyping array may provide technical assistance for the identification of causative and credible single nucleotide polymorphisms (SNPs) that affect the chicken RFI [15].

The transcriptome is a full set of the RNAs transcribed in the cell at a certain developmental phase or under a specific stress condition and possesses spatial and temporal expression characteristics [16]. RNA sequencing can be a gateway to success in identifying differentially expressed genes (DEGs), discovering alternative splicing events, and conducting studies on gene evolution [17–20]. Previous studies showed that the RFI was so complex that all relevant genes might not operate in a single physiological pathway [21, 22].

The present study combined GWAS with transcriptome sequencing to identify distinct genes or regions that affected the chicken RFI. Our results lay the foundation for improvements in energy efficiency and reductions in feed cost.

## Results

### Growth characteristics and genetic parameters

**Fig. 1:**
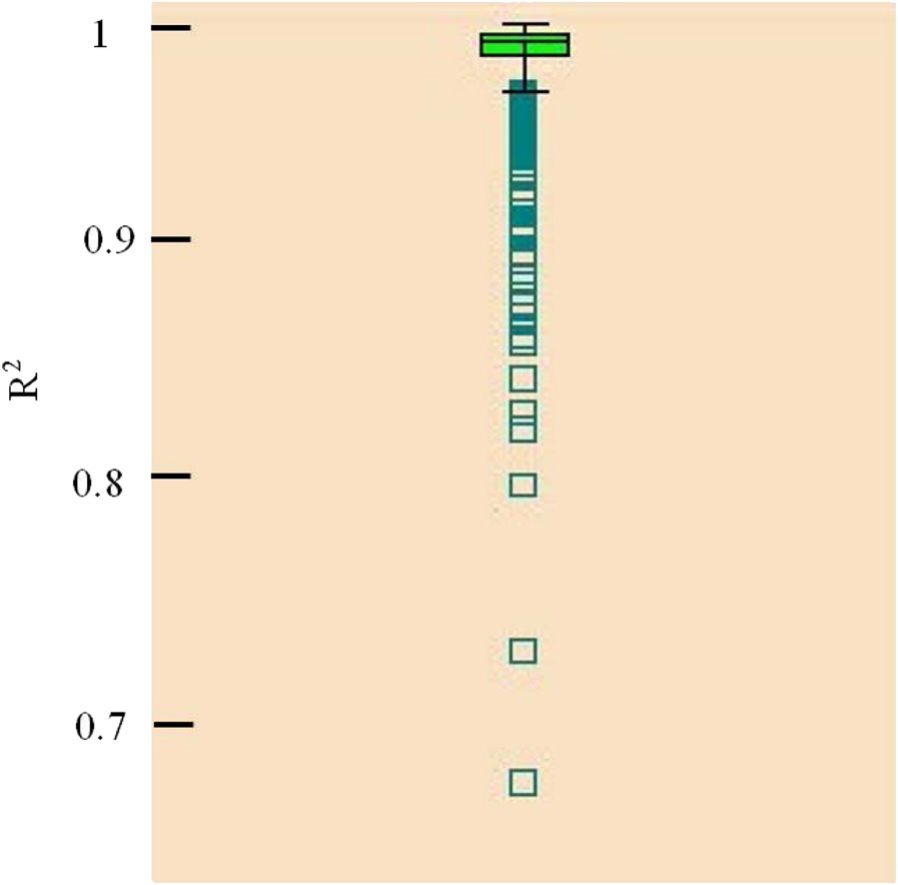
Distributional R^2^ for regression of body weight against the day of the test for each chicken. The vertical ordinate denotes the R^2^ for the growth rate

The pure line N301 belongs to the dwarfism yellow-plumage chicken with a medium growth speed. At the beginning of the feeding trial (44 d of age), the average body weights of the male and female chickens were 794 (±124) g and 760 (±98) g, respectively. When the trial was finished (83 d of age), the male chickens reached 1913 (±230) g and the female chickens reached 1780 (±160) g. The average daily feed intakes (ADFIs) of the male and female chickens recorded by the electronic feeding station were 109 (±15) g and 102 (±15) g, respectively. The linear regression equation well described the changes in body weight at different ages. The average R^2^ across all birds was 98.4 %. Only four birds had an R^2^ lower than 80 % (Fig. 1), suggesting that the estimates for average daily gain (ADG) and mid-test metabolic body weight (MMBW) for each bird were reliable.

The test of fixed effects showed that the gender effect on RFI was not significant (*P* > 0.1), but the pen effect was significant (*P* < 0.01). One explanation for this result may be that the chickens were already separated based on gender when they were arranged in different pens, thereby turning the nested gender effect into the pen effect. Next, we compared the growth traits between the birds with the highest- and lowest-ranked RFIs (Table 1). No significant difference was found for any growth trait, which was similar to the results obtained in cattle [23]. However, the ADFI for the lowest RFI-ranked birds was 39 g lower than the ADFI for the highest RFI-ranked birds (*P* < 0.01).

**Table 1:** Growth characteristics (average ± S.E.) of 10 % of the chickens with the lowest and highest residual feed intake rankings

Traits^a^	Lowest 10 %	Highest 10 %	*P*-value^b^
RFI during 44–83-d old (g)	−17.40 (±0.45)	20.16 (±0.56)	/
ADFI (g)	87.32 (±0.90)	126.10 (±1.18)	<0.01
Body weight at 44-d old (g)	771.60 (±9.31)	770.62 (±10.78)	0.94
Body weight at 83-d old (g)	1832.92 (±16.07)	1858.03(±16.07)	0.35
ADG (g)	27.8 (±0.31)	27.98 (±0.46)	0.75
MBW (g)	1318.3 (±11.69)	1335.0 (±15.12)	0.38
FCR during 44–83-d old	3.31 (±0.03)	4.76 (±0.09)	<0.01

^a^ *RFI* residual feed intake, *ADFI* average daily feed intake, *ADG* average daily gain, *MBW* mid-test body weight, *FCR* feed conversion rate

^b^ *P*-value obtained by *t*-test

The heritability estimate for the RFI from 44 to 83 d of age was 0.35 (Table 2), which fell into the 0.21 to 0.49 range reported for other populations [1, 2]. This result implied that the RFI was under a moderate level of genetic control. Phenotypic RFI selection provides a method to reduce feed intake without affecting either the ADG (r_p_ = 0.03) or MMBW (r_p_ = 0.01). However, RFI selection had a few consequences over the ADG if only the genetic contribution was considered (r_g_ = 0.27), which was consistent with previous reports [1, 2, 24]. The positive genetic correlation between RFI and FCR found in our population (r_g_ = 0.75) was higher than the correlation reported in other domestic animals [24, 25].

**Table 2:** Genetic parameter estimation for the growth and feeding traits

Traits^c^	No.^d^	Heritability (±S.E.)	RFI during44-83-d old (g)
RFI during44-83-d old (g)	1158	0.3542 ± 0.0701	/
ADFI (g)	1158	0.3950 ± 0.0749	0.7266^a^/0.7544 ± 0.0718^b^
Body weight at 44-d old (g)	1158	0.5598 ± 0.0848	−0.0293/−0.2059 ± 0.1462
Body weight at 83-d old (g)	1158	0.3683 ± 0.0765	0.0247/0.0314 ± 0.1649
ADG (g)	1158	0.3186 ± 0.0723	0.0286/0.2725 ± 0.1626
MMBW (g^0.75^)	1158	0.4706 ± 0.0821	0.0126/−0.0233 ± 0.1563
FCR during 44–83-d old	1158	0.2159 ± 0.0594	0.6558/0.7465 ± 0.0902

^a^Pearson correlation

^b^Genetic correlation (average ± S.E.)

^*c*^ *RFI* residual feed intake, *ADFI* average daily feed intake, *ADG* average daily gain, *MBW* mid-test body weight, *FCR* feed conversion rate

^d^ Number of samples for estimating heritability

### Genome-wide association study

**Fig. 2:**
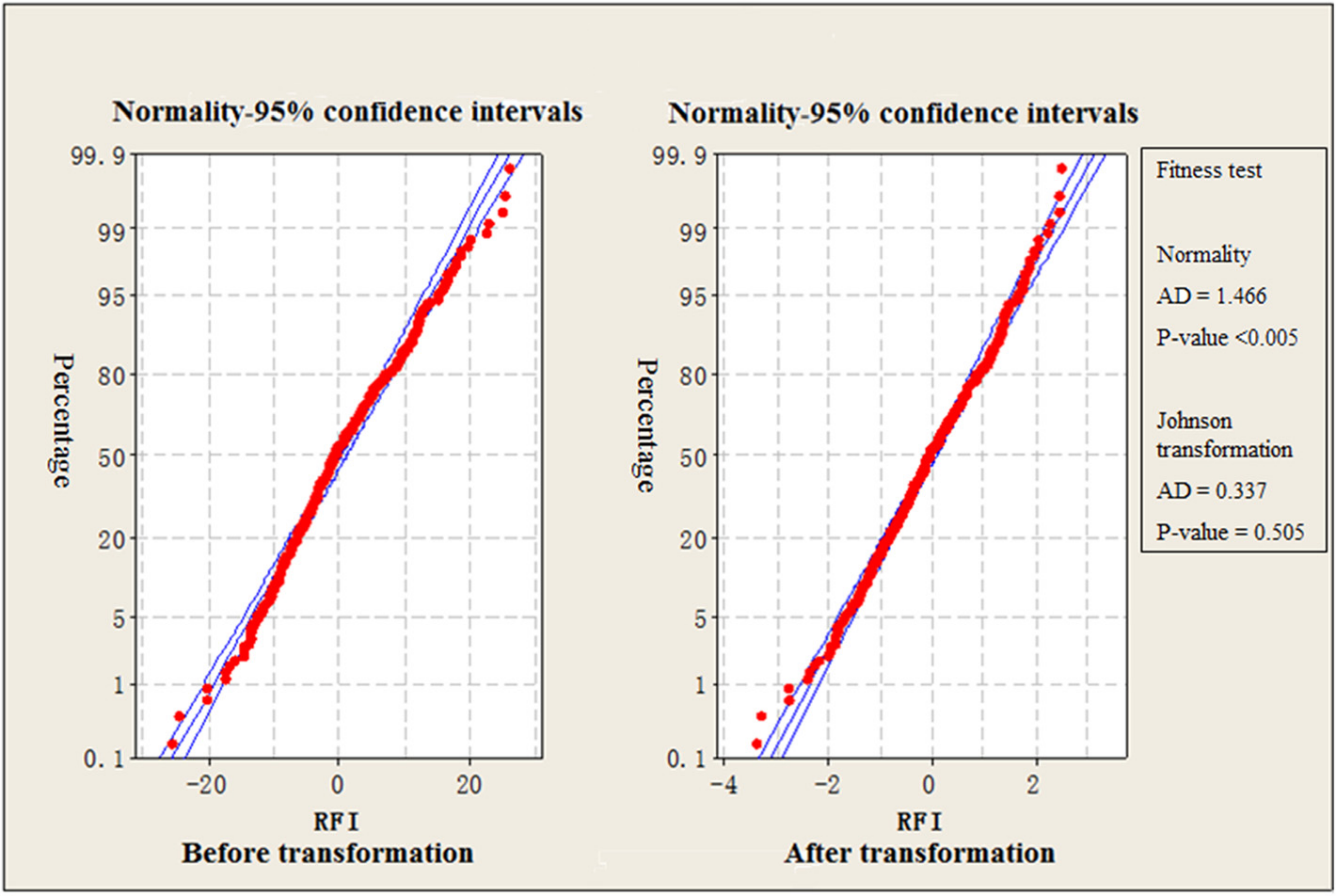
Probability graph of residual feed intake

The original RFI values calculated from 426 individuals were examined for compliance with normality prior to the GWAS using the Anderson-Darling test. The results showed that the initial distribution deviated from the normal distribution (*P* < 0.01) (Additional file 1: Table S1). After Johnson transformation, the phenotypic data were subjected to statistical analysis (Fig. 2).

**Fig. 3:**
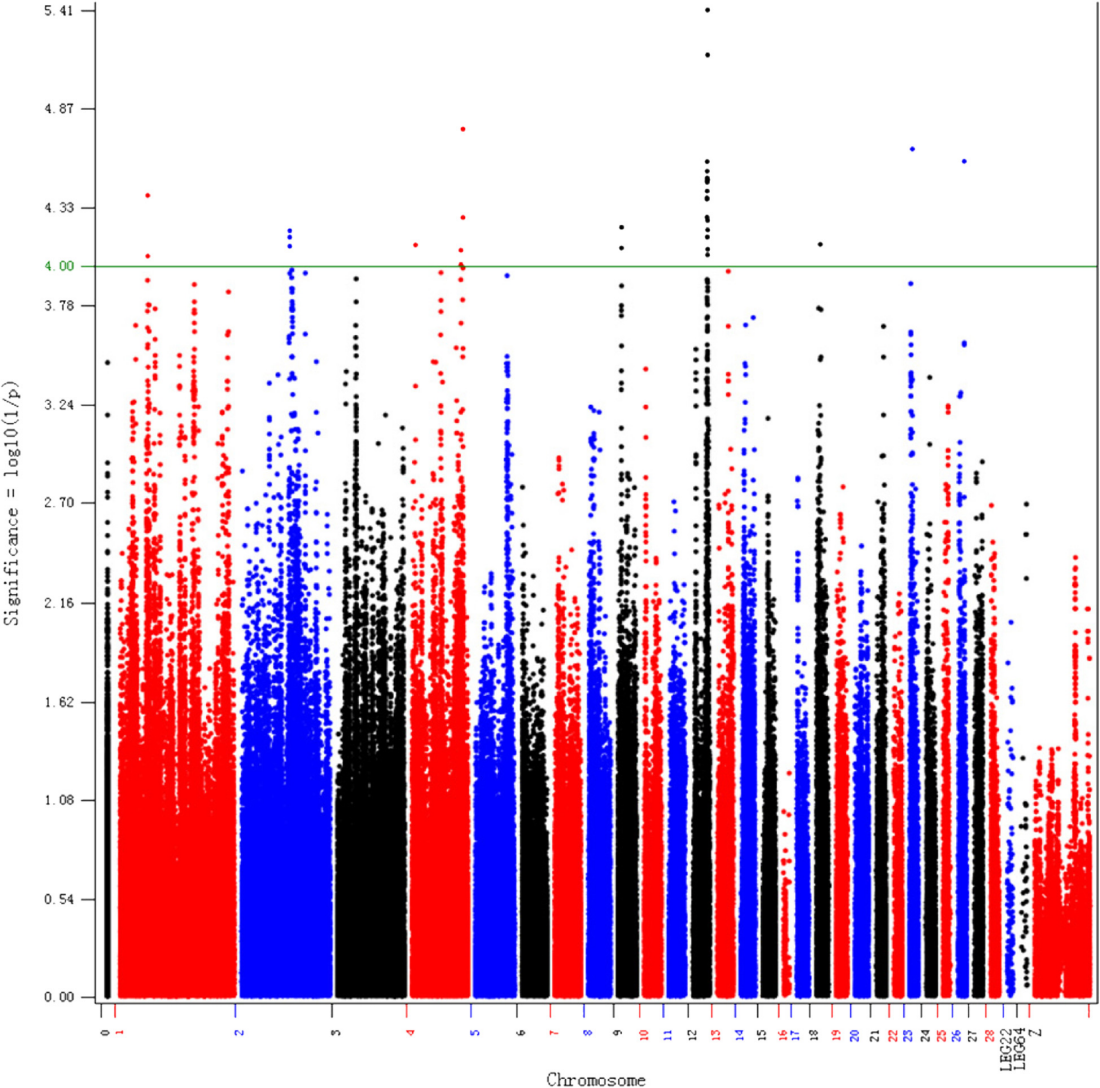
Manhattan plot of the SNP additive effects on residual feed intake (RFI) in chickens. The *green line* was the genome-wide significance threshold (10^−4^)

Because the kinship of all tested individuals could not be entirely eliminated when the IBS matrix was introduced into the mixed model, the genomic control inflation/deflation parameter (λ) was introduced [26]. In total, 32 SNPs were significantly associated with the RFI with *P*-values below the threshold (10^−4^) after correction by a λ equal to 1.005 (Fig. 3); these SNPs accounted for 53.01 % of the additive genetic variance. These significant points corresponded to 13 known genes and one unannotated gene in the chicken genome: *SLC17A8*, *COBL*, *PCDH19*, *JAKMIP1*, *ZFYVE28*, *PPP1R7*, *SEPT2*, *RYBP*, *PDZRN3*, *CHL1*, *UTS2R, ZMPSTE24*, *SYT6* and *LOC101749255* (Table 3).

**Table 3:** SNPs significantly associated with RFI

Locus	Chr	Position^a^	Effect^b^	*P*-value^c^	MAF^d^	Var(%)^e^	Nearest gene	SNP-gene relation
rs313001261	1	47140273	0.35	4.08E-05	0.36	9.52	SLC17A8	UTR-3
rs14820118	1	47142175	0.34	8.75E-05	0.35	8.53	SLC17A8	downstream
rs313802356	2	81015541	−0.44	6.90E-05	0.16	2.24	COBL	UTR-3
rs316977875	2	81047713	−0.44	6.37E-05	0.16	2.24	COBL	intron
rs312370583	2	81093795	−0.44	7.73E-05	0.16	2.42	COBL	intron
rs312326618	4	5197844	−0.3	7.60E-05	0.44	4.8	PCDH19	intron
rs316570003	4	78962129	0.38	8.13E-05	0.22	9.71	JAKMIP1	intron
rs14494646	4	79000778	0.44	9.74E-05	0.13	6.93	JAKMIP1	upstream
rs312925954	4	82293366	0.45	1.76E-05	0.16	4.52	ZFYVE28	intron
rs16445177	4	82306738	0.4	5.39E-05	0.18	4.29	ZFYVE28	intron
rs312767762	9	4440306	−0.53	7.90E-05	0.1	8.43	PPP1R7	intron
rs16663153	9	4507514	−0.51	6.10E-05	0.11	8.13	SEPT2	downstream
rs14046165	12	16386012	0.33	4.20E-05**	0.38	4.15	RYBP	intron
rs312899555	12	16393667	0.33	5.37E-05**	0.31	2.63	RYBP	intron
rs313947867	12	16400923	0.33	4.28E-05**	0.31	2.63	RYBP	intron
rs317049993	12	16423468	0.32	3.39E-05**	0.4	4.38	RYBP	upstream
rs315004580	12	16640380	0.32	3.00E-05*	0.47	5.54	PDZRN3	intron
rs315162282	12	16648446	0.32	3.27E-05*	0.47	5.54	PDZRN3	intron
rs317287197	12	16660649	0.32	2.66E-05*	0.47	5.54	PDZRN3	intron
rs316098097	12	16673403	0.32	3.86E-05**	0.47	5.54	PDZRN3	intron
rs314131263	12	16681057	0.32	3.45E-05**	0.47	5.62	PDZRN3	intron
rs14046530	12	16758193	−0.32	6.89E-05**	0.49	4.54	PDZRN3	upstream
rs315238546	12	17083942	0.3	5.60E-05**	0.41	5.94	LOC101749255	downstream
rs315693318	12	17089689	0.3	8.62E-05**	0.41	5.94	LOC101749255	downstream
rs315157887	12	17160768	0.37	3.92E-06**	0.38	7.41	LOC101749255	upstream
rs317584843	12	17195875	0.32	6.31E-05**	0.3	5.22	CHL1	upstream
rs314285248	12	17230860	0.33	6.92E-06**	0.46	5.08	CHL1	intron
rs317278144	12	17239654	0.35	3.34E-05*	0.25	7.47	CHL1	intron
rs317188563	12	17260556	0.28	8.02E-05**	0.5	1.88	CHL1	intron
rs316897066	18	3599249	0.28	7.54E-05	0.44	5.18	UTS2R	downstream
rs315285389	23	1733290	−0.38	2.27E-05*	0.24	3.39	ZMPSTE24	upstream
rs315491506	26	3777283	−0.38	2.65E-05	0.21	9.28	SYT6	intron

^a^Physical position

^b^Additive effect of allele B (minor allele)

^c^ *P*-value corrected for inflation factor λ, *FDR ≤10 %, ** FDR ≤5 %

^d^Minor allele frequency

^e^The proportion of the phenotypic variance accounted by the SNP

These SNPs were widely distributed on eight chicken (*Gallus gallus*) chromosomes (GGA). GGA18, GGA23 and GGA26 possessed only one significant SNP each. Two of the significant SNPs were located in the 3’ untranslated region (UTR) and downstream of *SLC17A8* on GGA1, two were located within a 4.4–4.6 Mb region on GGA9, three were located within a 81.0–81.1 Mb region on GGA2, five were located on GGA4. Notably, more than half of the total significant SNPs were located within a 1 Mb narrow region (16.3–17.3 Mb) on GGA12.

**Fig. 4:**
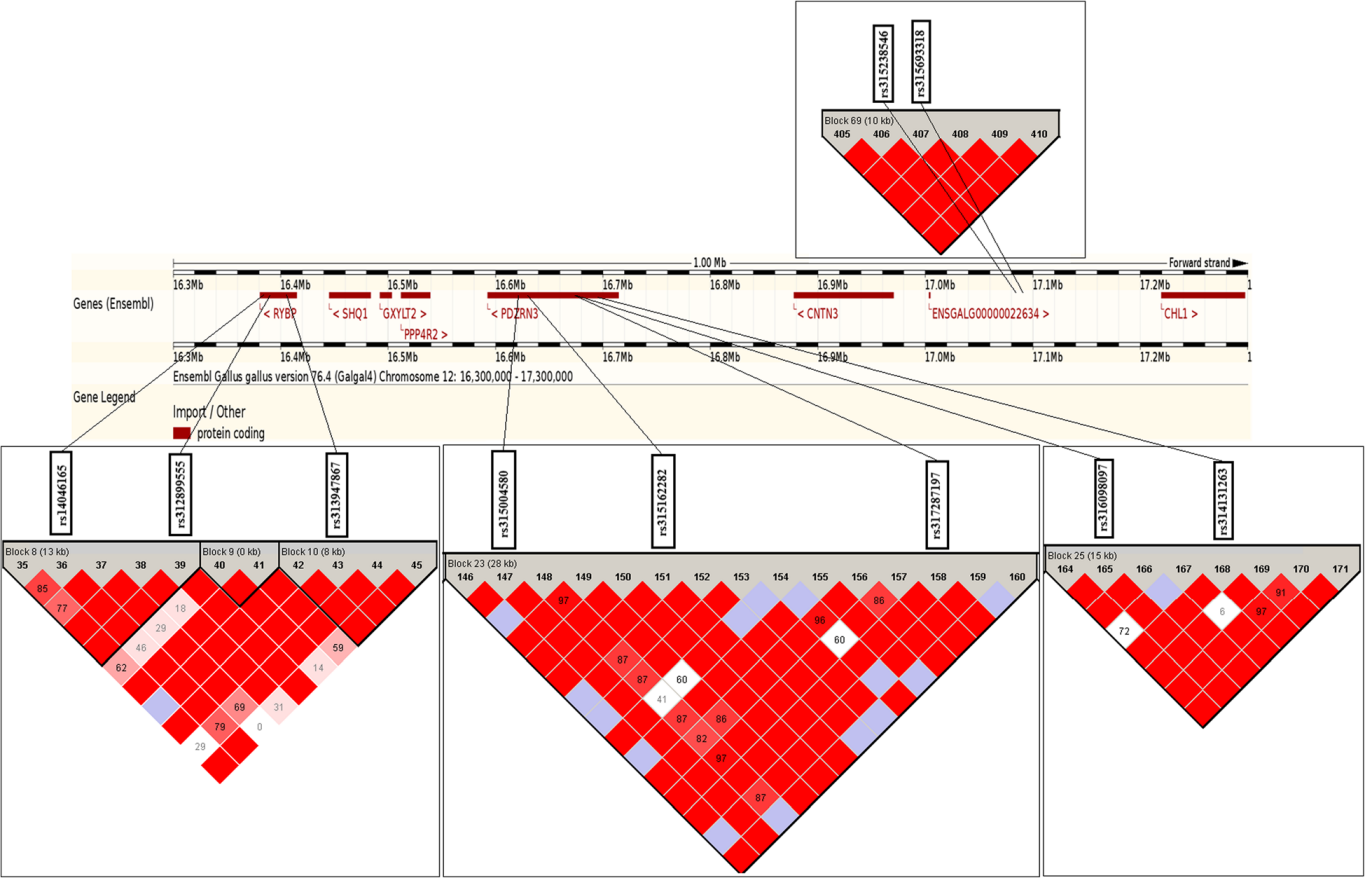
Ensembl genes on the region from 16.3 to 17.3 Mb on chicken chromosome 12 and haplotype blocks constructed using significant RFI-related SNPs

This narrow region contained 555 tested SNPs around eight genes. We observed 99 haplotype blocks extending from 0 to 64 kb (Fig. 4). The significant SNPs were entirely located within the blocks with the exception of SNP rs317049993. Three significant points in the *RYBP* introns (rs14046165, rs312899555, and rs313947867) had identical additive effects because they were in a state of complete linkage. Six haplotypes were found in a block determined by the SNPs rs14046165 and rs312899555; “GATGA” occurred more commonly in the other three non-significant SNPs with a frequency of 31.2 %. Four haplotypes were found in another block determined by the SNP rs313947867; “GGGC” was the most common haplotype in the other three non-significant SNPs with a frequency of 35 %. Five significant SNPs in complete linkage were found in the introns of *PDZRN3*, although they were positioned in two different blocks.

The proportion of phenotypic variance explained by rs316570003 in the intron of *JAKMIP1* was highest among the significant SNPs. Because the original RFI variable was transformed into the normal distribution data, the increment of the additive effect accompanied by the minor allele could not accurately reflect its impact on the change in RFI. In contrast, the direction of the additive effect could be used to judge whether the minor allele would be beneficial to the RFI. The results showed that nine wild-type mutations were in favor of decreasing the chicken’s RFI value.

**Fig. 5:**
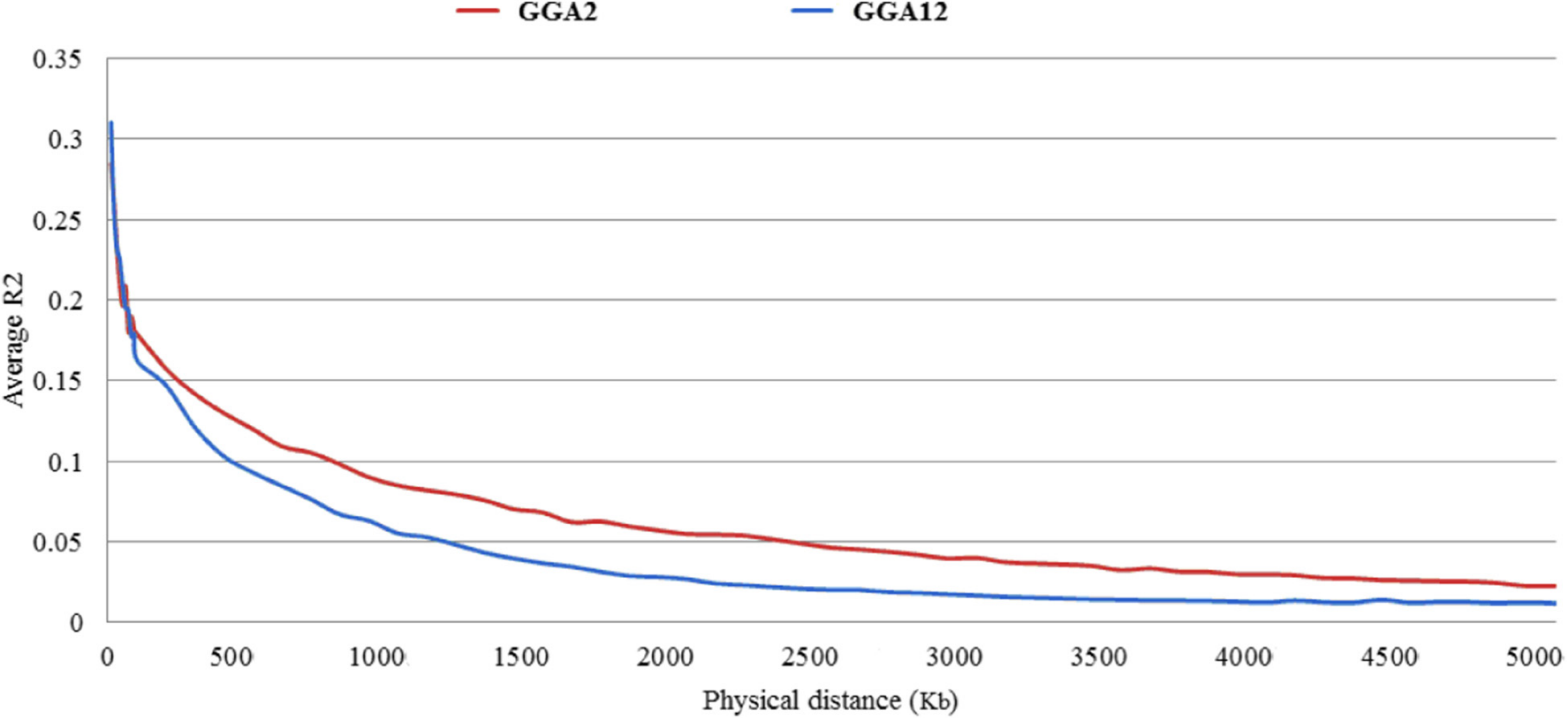
Relationship between the physical distance and LD values between SNP markers in the quality chickens used in the present study

The chip used in the present study had a very high density. The average interval between two SNPs was 1.8 Kb, which resulted in little chance for recombination among neighboring SNPs gathered in a limited range of the chromosome. Linkage disequilibrium (LD) analysis was performed to determine the extent to which the causative SNP could generate linkage to the significant SNP. The pairwise LD measured by *R*^2^ values for the present population was calculated for GGA12, where the most significant SNPs were concentrated. The results showed that the LD level was high (*R*^2^ = 0.3) at short distances, slightly decreased (*R*^2^ = 0.2) when the distances increased to 40–60 kb and low (*R*^2^ ≤ 0.15) when the distance increased to 80–100 kb. Similar trends were found in GGA2 (Fig. 5). Therefore, 50 kb was accepted as a reasonable distance to cause moderate LD between two SNPs, and we captured all genes distributed on the 50 kb flanking regions of the significant SNPs for GO analysis.

The results from the first level classification revealed that 45 GO terms in molecular function, 40 GO terms in biological process and 27 of GO terms in cellular component corresponded to the input genes (Additional file 2: Table S2). A deeper analysis was conducted in the molecular function GO terms to obtain more information. Protein binding (12.5 %) was reflected by eight different genes and might be the major molecular function that differed in RFI-divergent birds, followed by zinc ion binding (10 %), calcium ion binding (7.5 %), and ATP binding (5 %). Protein binding was significant, including the synthesis of a peptides from amino acid molecules and the formation of a high-grade protein structure by combining polypeptide chains. This result indicated that the difference in protein binding was associated with the rapid growth of the experimental birds, specifically muscle development. The chief role of the calcium ion was to compromise potential on two sides of the cell membranes and to maintain muscle contraction and relaxation, indicating that differences in motor skills would occur in RFI-divergent birds. Therefore, differences in ATP binding should not be overlooked. ATP is the primary source of fuel for every biochemical and physiological process and will induce metabolic disorders in the event of an exception.

### RNA sequencing of the transcriptome

#### Read mapping

RNA sequencing was performed in four individuals, as follows: two chickens with the highest RFI values (H1 and H2) and two with the lowest RFI values (L1 and L2). Among the four samples, H2 had the lowest mapping rate (total mapped reads/total reads) of approximately 88.4 % compared to the rates above 90 % for the other three samples (Table 4). Some of these reads had more than one matching sequence in the genome; these reads were removed from the subsequent analysis. The clean reads were evenly distributed across all chromosomes. Based on the annotation analysis of the clean reads mapped to the genome, we found that 61 % of these reads were derived from exons, 32.62–35.56 % were from intergenic regions, and only a small number were derived from splicing and intronic regions.

**Table 4:** Statistics for read mapping

Sample name	L2	H1	H2	L1
Total reads^a^	25,313,336	25,731,202	20,249,350	28,438,634
Total mapped^b^	22,702,806 (89.69 %)	23,216,653 (90.23 %)	17,916,485 (88.48 %)	25,601,368 (90.02 %)
Multiple mapped^c^	681,109 (2.69 %)	690,852 (2.368 %)	575,647 (2.84)	695,892 (2.45 %)
Uniquely mapped^d^	22,021,697 (87.00 %)	22,525,801 (87.54 %)	17,340,838 (85.64 %)	24,905,476 (87.58 %)
Reads map to “+”^e^	11,365,825	11,622,304	8,966,742	12,813,376
Reads map to “−”^f^	11,336,981	11,594,349	8,949,743	12,787,992

^a^Total reads: the number of clean reads

^b^Total mapped: the number of clean reads that could be mapped to the chicken genome

^c^Multiple mapped: the number of clean reads corresponding to a plurality of locations in the genome

^d^Uniquely mapped: the number of clean reads corresponding to a unique location in the genome

^e^Reads map to “+”: reads mapped to the sense strand

^f^Reads map to “−”: reads mapped to the antisense strand

#### DEG analysis

Four samples were classed into two groups based on their RFI rankings prior to the DEG analysis. The average expression of identical genes in the high-RFI group was compared with the low-RFI group to screen for DEGs. Biological replicates of the expressed genes had a high correlation coefficient (0.936) between the high-RFI group and low-RFI group (Additional file 3: Figure S1). As a generalized representation index, RPKM (reads per kilobase of transcript per million mapped reads) was used to denote each transcript’s expression quantity [27]. After bioinformatics assessment and comparison, 119 differentially expressed transcripts were identified between the high- and low-RFI groups that corresponded to 116 known genes. A total of 74 of the DEGs were up-regulated in the low-RFI group and the rest were down-regulated compared with high-RFI group (Additional file 4: Table S3). Interestingly, three genes (*CCKAR, LOC395159, and miR-122-1*) were only expressed in the low-RFI group. The expression of *miR-6705* was significantly lower in the low-RFI group than in the high-RFI group with a fold change of eight. In contrast, the expression of *PCK1* was significantly higher in the low-RFI group than in the high-RFI group with a fold change of 39.

MiRDB (http://mirdb.org/miRDB/) and TargetScan (http://www.targetscan.org/) are two effective websites for microRNA (miRNA) target prediction. Based on their prediction results, *miR-122-5p* and *miR-122-3p* were predicted to act on 693 target genes. Three targets (*COL3A1, PER2,* and *CAV1*) were found to be DEGs. However, these genes were up-regulated in the low-RFI group, which was not consistent with the negative regulatory interaction between the miRNA and its target. *MiR-6705-5p* was predicted to act on 704 target genes. Nine targets (*LPL, GLCCI1, COL3A1, B3GNT2, ABHD17B, CAV1, RHOBTB1, CHAC1,* and *LUM*) were found to be DEGs. Except for *LPL*, the other eight genes were up-regulated in the low-RFI group, indicating they might be negatively regulated by *miR-6705-5p*. Of note, the expression of *COL3A1* and *CAV1* could be impacted by both the *miR-122-1* and *miR-6705*.

Hepatic *PCK1* expression was drastically increased by 28-fold compared to its normal expression level. Similarly, the subcutaneous, intercellular, and abdominal fat contents were increased when a simple orchiectomy was implemented in capons, implying negative regulation of hepatic *PCK1* expression and abdominal fat content by testosterone [28].

## Discussion

In Koch’s model, b_2_BW^0.75^ is normally used as metabolic energy (ME) for maintenance. However, b_2_BW^0.75^ is most likely to represent only the ME maintenance output for basal metabolism. To determine the maintenance requirement, two non-determinative factors (energy for environmental stress resistance and voluntary activity) should be considered simultaneously. In the present study, RFI was phenotypically independent of MMBW (r_p_ = 0.01) and ADG (r_p_ = 0.03), indicating that there was no difference in the use of the ME for maintenance of the basal metabolism and for growth between high and low RFI individuals [29]. In reality, high-RFI birds tried to replenish their energy by eating more; thus, the redundant energy might be exploited by stress or physical activity [30]. We speculated that the low-RFI birds had less governable overall energy to expend on stress and physical activity. In the present study, the low-RFI birds spent less time eating (r_g_ = 0.49) (data not shown), indicating that they reduced the ME demand at the expense of less physical activity related to feed intake.

Four significant SNPs on GGA1 and GGA9 in the present study did fall into the reported QTLs for chicken RFI [31]. However, only the significant SNPs on GGA12 passed chromosome-wise FDR threshold of 5 %, probably because of limited sample size resulting in low power to detect a QTL by testing the marker effect. Another possible cause was the low RFI phenotypic variability in sex-linked dwarf chicken used in the present study. The RFI values of 426 individuals ranged from −25.6 g to 25.6 g. The standard deviation of the RFI was simply 8.36 g, which was much less than that observed in normal meat-type chickens [2].

In the present study, we identified eight genes located in a 1 Mb narrow region, and almost all of the significant points in GGA12 were located in introns. The SNPs in the introns could play negative, positive, or bidirectional regulatory roles in gene expression and affect alternative splicing [32, 33]. *PDZRN3* was reported to exert a negative effect on lipogenesis. In mouse 3 T3-L1 preadipocytes, up-regulation of STAT5b and C/EBPβ was observed in response to PDZRN3 silencing, resulting in increased expression of PPARγ at both the mRNA and protein levels and the promotion of 3 T3-L1 cell differentiation into adipocytes [34]. As a candidate gene for human schizophrenia and mental deficiency, *CHL1* is relevant to learning behavior and reorganization of the frame of thinking. The CHL1-deficient mice displayed reduced enthusiasm for fresh food hunting and a blockage in social contact that was attributed to chaos in the neural circuits that connected the brain’s limbic system to the cerebral cortex [35]. *JAKMIP1* was of special concern because its intronic SNP rs316570003 explained the highest phenotypic variance. This gene was reported to be associated with microtubule polymers and to participate in cytoskeleton rearrangement, cell polarization, and intracellular trafficking [36].

These findings combined with the implications of the GO analysis provided hints that RFI-related genes improved energy utilization efficiency by adjusting cellular procedural activities, thereby assisting muscle and neural development and growth and eventually improving and enhancing energy efficiency.

Transcriptome sequencing helped identify the DEGs causing the diversity in the RFI phenotypes. In the absence of mRNA expression, we speculate that there should be a role for *CCKAR* in the constant energy use. Indeed, CCKAR was reported to be associated with appetite control [37, 38]. CCK is a hormone that causes gallbladder contractions and enables the promotion of trypsin secretion [39]. Central CCK could be motivated by a combination of endogenous CCK and its receptor CCKAR to trigger a feeling of satiety. Indeed, an increased feed intake was detected in CCKAR-deficient mice [38], and the risk variants of porcine *CCKAR* were significantly correlated with feeding traits [40]. The effect of the *CCK* gene might be weakened by a lack of its receptor in high-RFI individuals, resulting in increased feed intake.

The DEGs were consigned to six different gene-interaction networks predicted by IPA (Additional file 5: Table S4). The networks were more comprehensive and detailed when combined with the function of homologous genes from humans and mice. For this reason, they provided redundant information that could not be applied to chickens, such as cancer or cardiac failure; thus, the related gene functions were simply suggestive in poultry. However, these networks provided important clues to improve the energy balance from the perspectives of genetics, nutrition and bio-pharmaceuticals.

Low RFI selection made dramatic changes in some biological pathways possible (Table 5). For example, the cell death and apoptosis of tumor cell lines, activation of antigen presenting cells (APCs) and cell viability of kidney cell lines were decreased during low RFI selection. Additionally, chickens with low RFI were more prone to vascular lesions. Every change was a result of the mutual action of DEGs.

**Table 5:** Predictable changes in biological functioning based on DEG analysis in low-RFI individuals

Categories	Functions annotation	*P*-value	Predicted	Molecules
Cell Death and Survival	apoptosis of lung cancer cell lines	5.80E-06	↓	CAMP, EREG, G0S2, HSPA5, MCL1, RASD1, SERPINF1, TNFSF10
	apoptosis of tumor cell lines	2.08E-05	↓	AR, BCL2A1, CAMP, CAV1, COL18A1, CREM, CTSD, EREG, G0S2, HSPA5, IL18, MAOA, MCL1, NFKBIA, NR0B2, PLAU, RASD1, SERPINF1, TNFSF10, UCHL1
	cell death of lung cancer cell lines	3.21E-06	↓	CAMP, EREG, G0S2, HSPA5, MCL1, NFKBIA, RASD1, SERPINF1, TNFSF10
	cell death of tumor cell lines	2.75E-05	↓	AR, BCL2A1, BTG1, CAMP, CAV1, COL18A1, CREM, CTSD, EREG, FETUB, G0S2, HSPA5, IL18, MAOA, MCL1, NFKBIA, NR0B2, PLAU, PPAT, RASD1, SERP
	cellular degradation	3.77E-04	↓	AR, CACNA1D, CAV1, CREM, CTSD, LYZ, UCHL1
Cellular Assembly and Organization	formation of filaments	1.36E-03	↑	AR, CAV1, COL18A1, HSPA5, PTGDS, SERPINF1, TTR, TUBB
Organismal Development	development of body axis	1.99E-04	↑	ANGPTL3, CCKAR, COL18A1, CREM, CTSD, DIO3, GPR34, HHEX, HSP90B1, INSIG1, LUM, MAOA, NFKBIA, PLAU, SERPINF1
Immune Cell Trafficking	activation of antigen presenting cells	3.47E-04	↓	CAMP, GJB1, HNF4A, HSP90B1, IL18, LECT2, SERPINF1, TNFSF10
Renal and Urological System Development	cell viability of kidney cell lines	3.05E-04	↓	CAV1, HSP90B1, HYOU1, NFKBIA
Cardiovascular Disease	vascular lesion	2.94E-07	↑	ACAT2, CACNA1D, COL18A1, IL18, LPL, MGP, mir-221, NFKBIA, PLAU, TNFSF10

Apoptosis and APC viability warranted attention because they were tightly linked to immunity.

Apoptosis was effectively under the control of genes. When low-RFI birds suffer from Avian Leukosis Virus, Marek’s Disease or reticuloendotheliosis, related DEGs might be responsible for blocking the energy supply to tumor cells, thereby slowing apoptosis and accelerating tumor formation.

APCs are capable of ingesting and processing pathogenic microorganisms by phagocytosis or pinocytosis, which in turn produces peptide fragments that contain antigenicity domains [41, 42]. Decreased activation of APCs in low-RFI birds might cause a delay in the immune response or a reduced ability to kill pathogens. *HSP90B1* is an important immune protein that is involved in cell protection under heat stress. Increased HSP90B expression in response to heat stress causes various reductions in cell damage and even repairs damaged proteins [43]. The reduced *HSP90B1* expression in low-RFI birds signified a weaker ability to resist the effect of environmental factors compared to high-RFI birds.

**Fig. 6:**
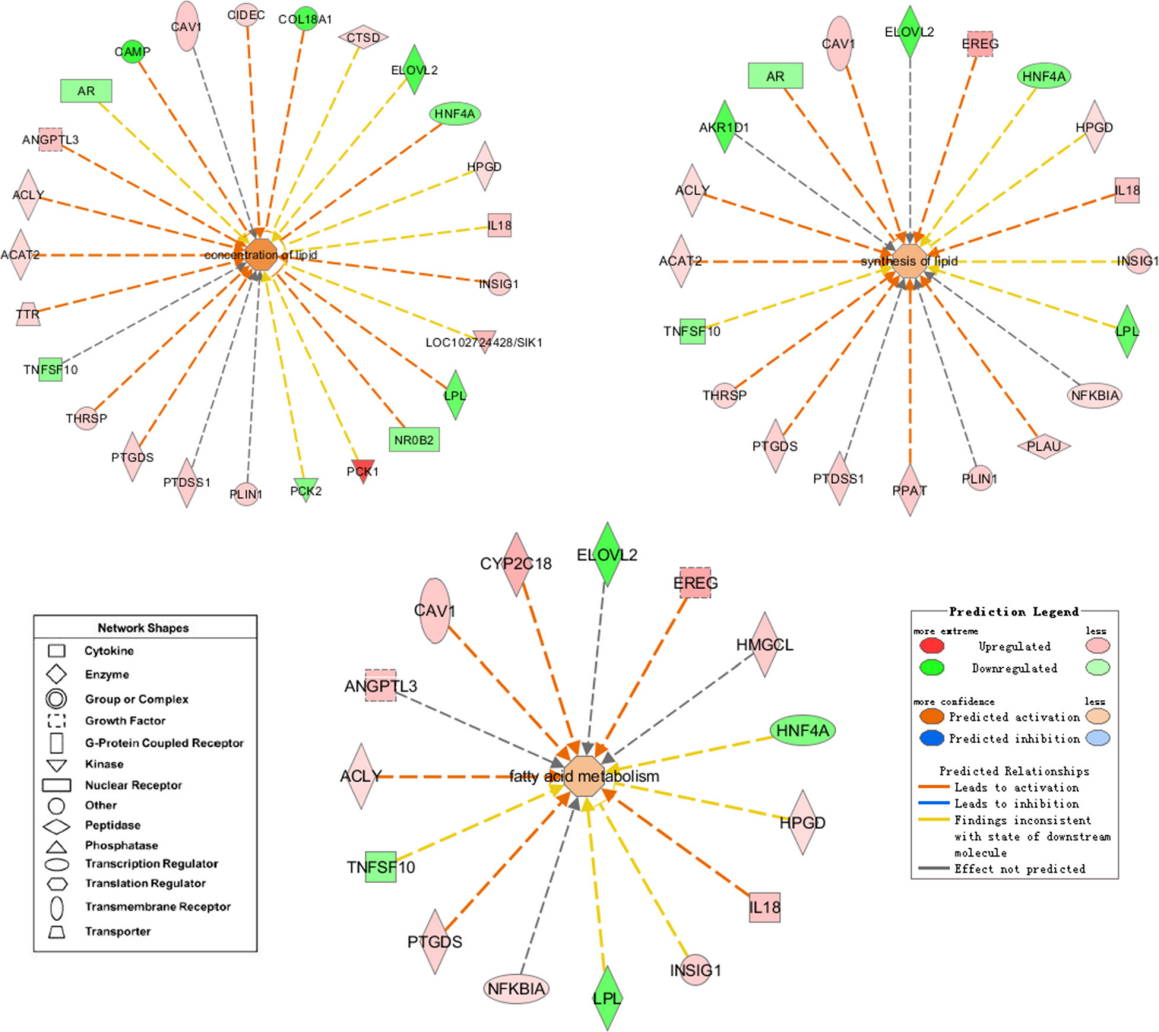
Predictable changes in fat metabolism based on DEG analysis in low-RFI individuals

Additionally, the changes in some DEGs were found to function in the regulation of fat metabolism (Fig. 6). For low-RFI birds, 13 DEGs were predicted to facilitate the concentration of lipids, 10 were predicted to play a pivotal role in the activation of lipid synthesis, and 5 were associated with the activation of fatty acid metabolism. Although a few DEGs had inhibitory effects, the above three specific physiological functions seemed to be enhanced overall. The results in the present study showed that low-RFI birds could achieve greater synthetic metabolism of lipids compared to high-RFI birds, whereas the results of another study showed that they displayed weaker catabolic abilities [44]. The faster anabolism of fat than catabolism in low-RFI birds might cause abnormal storage of fat and a decrease in heat release.

Among the lipometabolism-related DEGs, *PCK1* was particularly noteworthy because it was remarkably increased in the low-RFI group. The *PCK1* gene is associated with obesity, insulin resistance, and type II diabetes in mammals [45–47]. Abdominal fat content was assumed to be positively correlated with *PCK1* mRNA expression in birds [28]. Thus, high *PCK1* expression in low-RFI birds might signal a concurrent increase in the abdominal fat mass. Interestingly, *AR* expression was significantly lower in the low-RFI birds than in the high-RFI birds. The interaction of androgen with its receptor (*AR*) was closely related to bone metabolism in birds [48]. The AR in the cytoplasm enters the nucleus by integrating with androgen and then binding to the androgen responsive element, leading to the activation of androgen [49]. Low *AR* expression interferes with the positive physiological effect of androgen, leading to high *PCK1* expression.

A systematic analysis of the GWAS and RNA sequencing results was performed. The results showed there was no overlap of the genes within the 50 kb flanking region containing the significant SNPs detected by GWAS and any DEG detected by RNA sequencing. One explanation is that the actual linkage distance between the QTLs and the significant SNP is larger than 50 kb. However, we stopped measuring the RFI when the chickens were 83 d of age, whereas the liver sample collection was performed at 91 d of age. During this time, the RFI ranking may have slightly changed, resulting in a mismatch. Nonetheless, when the search scope was broadened [11] we still found that some potential genes within the 500-kb flanking region of the significant RFI-related SNPs in GWAS were identified as DEGs in chickens with divergent RFIs, including *MGP*, *HIST1H110*, *HIST1H2A4L3* and *OC3* near two SNPs (rs313001261 and rs14820118) in GGA1, *NR0B2* near the SNP rs315285389, *PER2* near the SNP rs16663153, *ST6GALNAC2* near the SNP rs316897066, and *G0S*2 near the SNP rs315491506. Notably, the concurrent genes involved in GWAS and RNA sequencing played critical roles in a wide spectrum of biological processes. Therefore, their relationships with RFI need to be verified.

## Conclusions

The heritability of RFI during the period from 44 to 83 d of age was moderate. The RFI was particularly affected by 32 significant SNPs that could explain 53.01 % of the additive genetic variance. Seventeen RFI-related SNPs were located in a 1 Mb region (16.3–17.3 Mb) of GGA12. This region was identified as a key candidate region affecting the energy utilization efficiency of chickens because it contained genes associated with lipogenesis, social behavior, and immunity. Although they obtained less metabolic energy, low-RFI birds maintained normal growth that was comparable to that of the high-RFI birds by allocating less energy to cell activity, the immune response, and physical activity in relation to eating and thus optimizing the use of the limited resources. Nevertheless, low-RFI selection cannot mobilize spare energy to withstand environmental stress and causes potential health risks that should not be ignored. At the same time, the low-RFI birds tended to become fatter.

## Methods

### Animals and measurement of feeding traits

The yellow-plumage dwarf chicken line N301 was used in the present study. Chicks with common characteristics of incompetent physical conditions (chicks that were malnourished or crippled) were eliminated after birth. The chickens were raised in a closed house to control the temperature and illumination during the early growth stage (0–4 wk of age). At the end of week 4, electronic chips were placed below the jaw and in the middle of the wattles. Then, the chickens were transferred to a half-open vertical ventilation hoop house. A total of 620 male chickens were maintained in three fence-separated pens on one side of the house, and 538 female ones were maintained in three fence-separated pens on the other side of the same house. Each pen had 17 feeding stations and 5 hanging water fountains in a 20 × 6 square meter area. The animals were fed a diet containing 2837 kcal/kg ME and 200 g CP/kg during the early growth stage. Then, the diet was switched to a high-energy diet containing 2900 kcal/kg ME and 190 g CP/kg. Daily feed intake and body weight were recorded for each bird throughout the feeding trial from 44 to 83 d of age.

### Calculation of residual feed intake

The ADG and MMBW for each individual were estimated based on the traditional model that was previously presented [50] and applied [23]. Age was expected to be linearly related to body weight because the experimental birds were in a period of rapid growth. Thus, their relationship was in compliance with the following equation: *BW =* μ *+ a × DOT + e*, where μ was the intercept, was the regression coefficient that represented ADG, DOT was the day of the test, and e was the residual. The mid-test body weight (MBW) was the predicted body weight on day 21 of the test, whereas MMBW was the MBW raised to the 0.75th power. ADFI was the mean of the daily feed intake throughout the 40 d of the experiment. The RFI was calculated from the subsequent model when all of the above parameters for each bird were estimated as follows: *ADFI = b0 + b1 × MMBW + b2 × ADG + RFI*, where b0 was the intercept, b1 and b2 represented the partial regression coefficients for MMBW and ADG, respectively, and RFI was the residual of the model.

To better understand the difference in the growth and feeding characteristics among individuals with divergent RFI values, data from the 10 % highest- and 10 % lowest-ranked RFI birds were pooled and t-tests were performed on the variables. Given the uncertainty of the gender or pen impact on the RFI, the RFI was corrected once the fixed effect was significant (*P* < 0.01) after it was calculated for the entire population.

The restricted maximum likelihood method implemented by the DMU package was used to obtain estimates of the phenotypic and genetic (co)variance and heritability [51]. The basic model was: y = Xb + Za + e, where y was the vector of the observations, a was the vector of the animal additive genetic effect, b was the vector of the fixed effects, including gender (two levels) and pen (six levels), e was the vector of random residuals, and X and Z were the incidence matrices. 1158 individuals were included.

### SNP genotyping and quality check

Blood samples from 435 male chickens randomly selected from the entire population were collected for DNA extraction using the EZNA Blood DNA Kit (Omega Biotek, Doraville, GA). The DNA samples were qualified and standardized into a final concentration of 50 ng/μl. Genotyping was performed with the Affymetrix 600 K genotyping array by Biotechnology Corporation (Shanghai, China). The genotyping quality control was evaluated in the GenABEL package in the R software (Additional file 6: Figure S2). A total of 415,154 SNPs and 426 individuals were involved in the final GWAS.

### Statistical analysis of the single marker GWAS

A general linear mixed model was used for the SNP-phenotype association as follows: Y = Xb + Sa + Zu + e, where Y was the vector of the RFI values after normal transformation and b was the estimator of the fixed effect. Because the pen effect did not exert a significant influence on the normal-transformed RFI in the male chickens, the fixed effects were ignored in this model. a was the SNP substitution effect and u was the random additive genetic effect following the multinomial distribution u ~ N (0, ${\Phi \upsigma}_{\mathrm{G}}^2$); here, Φ was the relationship matrix and ${\upsigma}_{\mathrm{G}}^2$ was the polygenetic variance. The genomic kinship matrix was used for the adjustment for population structure due to its better ability to estimate true covariance between individual genomes [52]. S and Z were the incidence matrices for a and u. e was a vector of residuals with a distribution of N (0, ${\mathrm{I}\upsigma}_{\mathrm{e}}^2$); here, ${\upsigma}_{\mathrm{e}}^2$ was the residual variance.

### The level of significance

Because sub-structures could still exist in the population, the *P*-value was corrected using the genomic control inflation/deflation parameter (λ) as a simple and rational option to solve the family data issue [26]. The significant threshold *P*-value after λ correction was 10^−4^, based on a hint by The Wellcome Trust Case Control Consortium and some articles [53, 54]. A chromosome-wise false discovery rate (FDR) method was used for verifying significant SNPs. The *p*-values of each SNP were sorted in ascending order, and the following formula was applied to obtain FDR for each SNP: mP(i)/i, in which m is the total number of SNPs, and P is the *p*-value of the ith SNP [55].

### Haplotype analysis

Linkage disequilibrium analysis was implemented in the region where multiple significant SNPs clustered using the Haploview software [56]. A pairwise strong LD was defined as the case when the one-sided upper and lower 95 % confidence limits of D’ exceeded 0.98 and 0.7, respectively. The block was constructed in cases when over 95 % of the informative SNPs in a region displayed a strong LD to identify potential regions of causal mutation for RFI [57].

### Phenotypic and additive variants analysis

The fraction of phenotypic variance explained by the significant SNPs was computed by the following model: *y =* μ *+ SNP + e*, where y was a vector of the RFI values, μ was the population mean, SNP was a vector of the genotypes in different individuals with regards to the significant SNP (fixed effect), and e was a vector of random errors. The proportion of the phenotypic variance explained by the SNP was reflected by the determination coefficient (R^2^) in the linear model. The proportion of additive variance explained by the SNP was computed in a similar manner; the only difference was that the phenotypic values were substituted for the breeding values.

### RNA extraction and RNA sequencing

Total RNA was extracted from the liver samples using the TRI reagent (Ambion, Applied Biosystems) according to the manufacturer’s instructions. The ND-2000 (NanoDrop Technologies) was used to verify the RNA integrity, and the Agilent Bioanalyzer 2100 (Agilent Technologies) was used to measure the concentration of the RNA samples. An RNA integrity number larger than 8.0 was considered to be acceptable for cDNA library construction.

Four samples (two with the highest RFI values and two with the lowest RFI values; Additional file 7: Table S5) were selected for cDNA library construction for RNA sequencing using the TRIzol reagent following the manufacturer’s instructions (Invitrogen). Purified mRNA was fragmented into 200–500-bp fragments and then reverse transcribed into cDNA. The samples were sequenced on a Genome Analyzer IIx (Illumina). The 2 × 100 bp sequencing strategy was adopted.

After sequencing, the generated raw reads were processed to clean reads by filtering low quality reads and adaptor dimers. After presenting the statistical distributions of the GC content and base quality, the filtered reads were mapped to the chicken reference genome using TopHat [58]. The parameters for mapping were as follows: reads-mismatches = 2 and reads-gap-length = 1. The clean reads were assembled and conjoined into contigs using Trinity (http://trinityrnaseq.github.io). The resulting contigs were connected into unigenes and annotated by ANNOVAR [59]. The expression of each gene represented by RPKM was estimated by the DEGseq package in R [60]. Differentially expressed transcripts or genes were identified when the threshold Benjamin *q*-value was below 0.05 (cut-off at a 5 % false discovery rate) and |log_2_ (fold change) | ≥1, respectively.

### Gene network construction

The DEGs were uploaded to the IPA database (http://www.ingenuity.com) for function and interaction analysis.

## Abbreviations

ADFI, average daily feed intake; ADG, average daily gain; APCs, antigen presenting cells; DEGs, differentially expressed genes; FCR, feed conversion rate; FDR, false discovery rate; GGA, chicken (*Gallus gallus*) chromosome; GWAS, genome-wide association study; LD, linkage disequilibrium; MBW, mid-test body weight; ME, metabolic energy; miRNA, microRNA; MMBW, mid-test metabolic body weight; QTLs, quantitative trait loci; RFI, residual feed intake; RPKM, reads per kilobase of transcript per million mapped reads; SNPs, single nucleotide polymorphisms; UTR, untranslated region

## Additional files

Additional file 1: Table S1. Normality test for residual feed intake. (DOC 28 kb) Additional file 2: Table S2. GO mapping for genes within the 50 bp flanking regions of the significant RFI-related SNPs. (XLS 31 kb) Additional file 3: Figure S1. Biological replicates of the expressed genes in the two RFI-divergent groups. (PNG 162 kb) Additional file 4: Table S3. Differentially expressed genes identified in RNA sequencing in divergent RFI individuals. (XLS 37 kb) Additional file 5: Table S4. Networks constructed using the differentially expressed genes predicted by IPA. (XLS 1284 kb) Additional file 6: Figure S2. The flow chart for genotyping quality control for the SNPs and individuals. (PNG 97 kb) Additional file 7: Table S5. Growth characteristics of the samples used for RNA sequencing. (DOC 27 kb)
