# Supplementary material for: Combination analysis of genome-wide association and transcriptome sequencing of residual feed intake in quality chickens
Source: BMC Genomics. 2016 Aug 9;17:594. doi: 10.1186/s12864-016-2861-5 (PMC4979145; doi:10.1186/s12864-016-2861-5)
Supplement: Additional file 1: Table S1. — Normality test for residual feed intake. (DOC 28 kb) [file 12864_2016_2861_MOESM1_ESM.doc]

Table S1 Normality test for residual feed intake

| Trait | N | Mean | SD | Nomality test | | |
| --- | --- | --- | --- | --- | --- | --- |
|  |  |  |  | Kurtosis | Skewness | AD test |
| RFI (g) | 426 | 0 | 8.36 | 0.23 | 0.29 | P < 0.01 |
