# Supplementary figures and images for: Combination analysis of genome-wide association and transcriptome sequencing of residual feed intake in quality chickens
Source: BMC Genomics. 2016 Aug 9;17:594. doi: 10.1186/s12864-016-2861-5 (PMC4979145; doi:10.1186/s12864-016-2861-5)

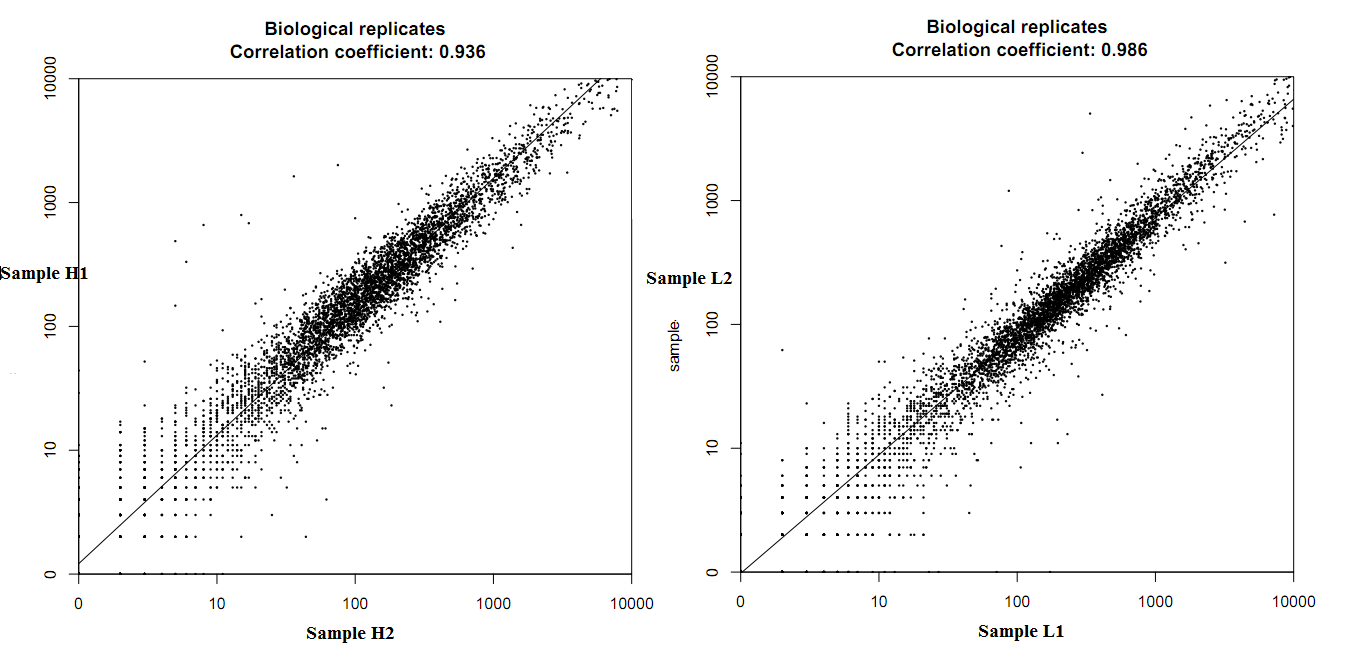

Supplement: Additional file 3: Figure S1. — Biological replicates of the expressed genes in the two RFI-divergent groups. (PNG 162 kb) [file 12864_2016_2861_MOESM3_ESM.png]

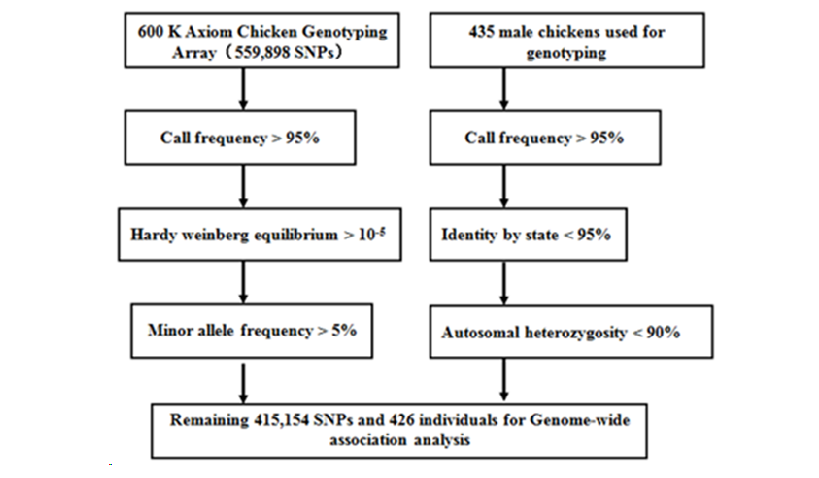

Supplement: Additional file 6: Figure S2. — The flow chart for genotyping quality control for the SNPs and individuals. (PNG 97 kb) [file 12864_2016_2861_MOESM6_ESM.png]
