# Supplementary material for: Combination analysis of genome-wide association and transcriptome sequencing of residual feed intake in quality chickens
Source: BMC Genomics. 2016 Aug 9;17:594. doi: 10.1186/s12864-016-2861-5 (PMC4979145; doi:10.1186/s12864-016-2861-5)
Supplement: Additional file 7: Table S5. — Growth characteristics of the samples used for RNA sequencing. (DOC 27 kb) [file 12864_2016_2861_MOESM7_ESM.doc]

Table S5 Growth characteristics of the samples used for RNA sequencing

| Pen^（1）^ | ID | ADFI | ADG | MBW | MMBW | RFI | IW | FW |
| --- | --- | --- | --- | --- | --- | --- | --- | --- |
| 3 | L1 | 89 | 29 | 1300 | 216.5 | -17.69 | 795 | 1803 |
| 3 | L2 | 89 | 29 | 1366 | 224.7 | -20.84 | 817 | 1840 |
| 2 | H1 | 134 | 31 | 1277 | 213.6 | 26.00 | 766 | 1810 |
| 3 | H2 | 130 | 26 | 1334 | 220.7 | 25.31 | 849 | 1768 |

(1) ID is abbreviation for identification number; ADFI is abbreviation for average daily feed intake (g); ADG is abbreviation for average daily gain (g); MBW is abbreviation for mid-test body weight (g); MMBW is abbreviation for mid-test metabolic body weight (g^0.75^); RFI is abbreviation for residual feed intake (g); IW is abbreviation for body weight at 44-d old (g); FW is abbreviation for body weight at 83-d old (g).
